# Enhanced Electrocatalytic Stability of Platinum Nanoparticles Supported on Sulfur-Doped Carbon using *in-situ* Solution Plasma

**DOI:** 10.1038/s41598-019-49194-x

**Published:** 2019-09-03

**Authors:** Oi Lun Li, Zhicong Shi, Hoonseung Lee, Takahiro Ishizaki

**Affiliations:** 1 School of Materials Science and Engineering, Pusan National University, Busan, 46241 Korea; 2 School of Materials and Energy, Guangdong University of Technology, Guangzhou, 510006 China; 3 Energy Environment Center Fusion Energy Team, Korea Marine Equipment Research Institute, Busan, 49111 Korea; 4 Department of Materials Science and Engineering, College of Engineering, Shibaura Institute of Technology, Tokyo, 135-8548 Japan

**Keywords:** Electrocatalysis, Synthesis and processing

## Abstract

The metal-air battery is a form of renewable energy generation technology that produces energy electrochemically and can address energy concerns in the near future. However, state-of-the-art Pt electrocatalysts often suffer from agglomeration or detachment from carbon supports under prolonged operation, eventually limiting the long-term utilization of metal-air batteries. In this work, Pt nanoparticles were deposited on sulfur-doped nanocarbon to increase its stability. We first synthesized sulfur-doped (S-doped) and pristine carbon as support materials via a plasma process, and thereafter loaded platinum (Pt) nanoparticles onto the S-doped and pristine carbon matrix. From a sintering test at 600 °C, the Pt nanoparticles supported on pristine carbon increased from 2.4 to 5.2 nm; meanwhile, the average size of Pt NPs supported on S-doped carbon only increased from 2.2 to 2.51 nm. From the electrochemical analyses, the mass activity of Pt on pristine and S-doped carbon supports decreased by 25% and 10%, respectively, after 1500 cycles. The results proposed that the sulfide C–S–C bond provided a strong platinum-S-doped carbon support interaction between the support materials and the loaded Pt nanoparticles. Thus, S-doped carbon supports can serve as a stabilizer of Pt nanoparticles to enhance their durability in the application of metal-air batteries and other electrochemical devices.

## Introduction

Electrochemical energy storage devices, including metal-air battery and fuel cell, are considered as strong candidates in future energy network^1–5^. However, the sluggish rate of oxygen reduction reaction (ORR) at the cathode has been a major challenge^6,7^. In order to increase the cell performance, platinum (Pt) or its alloy are often required as electrocatalyst^8,9^. Pt nanoparticles are normally supported on high surface-area carbon materials as state-of-the-art ORR electrocatalysts, yet these Pt nanoparticles often agglomerate or detach from the carbon supports after long operation times^10^. Heteroatom doping in carbon materials has been widely studied in materials science in past decades because their physiochemical properties can be significantly changed by simple manipulation of carbon nanostructures, which leads to expanding their applications^11–15^. In particular, sulfur-doped carbon has attracted much attention in the last few years because of its strong affinity to noble metal nanoparticles^16,17^. Ahmadi *et al*. have reported that Pt nanoparticles supported on sulfur-modified carbon nanotubes showed higher dispersion and narrow size distribution. Lee *et al*. reported a strong interaction between platinum nanoparticles and sulfur-containing carbon materials, and demonstrated a steady catalytic activity during durability test^18^. Since sulfur atom can form a strong covalent bond within the carbon surface, it is hard to extract them with chemicals and or decompose them by heating in vacuum up to 1000 °C. In the case of atmospheric conditions, sulfurized carbons showed good stability even after heat treatment of 1200 °C^19^. Thus, carbon supports functionalized with sulfur atoms might provide a new approach towards the stabilization of nanometer-sized Pt nanoparticles.

Sulfur-doped carbon can be divided into two categories: *in-situ* and post-treatment processes. The *in-situ* process is conducted by direct carbonization of sulfur-containing substances, including polymers or ionic liquids^20,21^. On the other hand, post-treatment is generally performed by impregnating sulfurizing gases or liquids onto the bulk carbonaceous materials or infiltrating sulfuric solid into porous carbon materials^20^. Various chemical bindings in sulfurized carbon materials are inevitably generated via conventional methods due to the complex treatments or heating process. In addition, chemical sulfur bindings are altered according to the types of sulfurizing materials and heating method. For instance, by impregnating with various sulfur sources (e.g. S, H_2_S^22,23^, CS_2_ and dimethyl disulfide^24,25^, SO_2_^26,27^, Na_2_S and polysulfide^28,29^) between temperatures of 150 to 800 °C, it is easy to form surface complexes such as C–S, S–S, C=S, S=O bond-containing groups or sulfide, hydrosulfide groups, sulfoxide, sulfone and thiophene^30^. In order to eliminate impurities and obtain high purities of purposed sulfur bindings, well-defined processes or additional steps like purifications, chemical or heat treatments are required. Another major drawback of post-synthesis includes the possible elimination of sulfur content in the form of hydrogen sulfide by heating at 700–800 °C in a hydrogen atmosphere^31^. Eventually, the sulfur content within the carbon matrix is too low to demonstrate the enhanced effect of sulfurized carbon materials as a durable stabilizer against nano-sized platinum nanoparticles.

Herein, an *in-situ* solution plasma (SP) process has been introduced as a new, simple and effective sulfur-doping method for controlling the chemical bonding state by adjusting various precursors^32–35^. The carbon matrix can easily dope with heteroatom similar to the molecular structure of its original precursor(s). The process has been utilized as a novel bottom-up synthesis route for nitrogen-doped, dual boron-nitrogen-doped, as well as other halogen-doped carbon catalysts. In this study, we firstly synthesized sulfur-doped carbon nanoparticles as support materials from thioanisole (TOAS) *via* the SP process and then loaded platinum (Pt) nanoparticles on the pristine and S-doped carbon matrix (Pt/TOAS). The thermal stabilization of Pt metal nanoparticles supported on TOAS (Pt/TOAS) was achieved against temperature treatment of 600 °C, which implies a combination of chemical interactions between the S-doped carbon surfaces and Pt nanoparticles. The chemical stabilization of Pt/TOAS under alkaline conditions was also investigated using multiple cyclic voltammetry (CV) tests.

## Materials and Methods

### Synthesis of pristine and S-doped carbon supports by plasma synthesis

**Figure 1:**
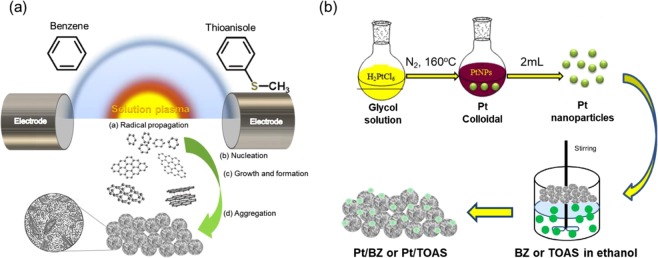
Schematic illustration of the formation mechanism of pristine carbon (BZ) and S-doped carbon particles (TOAS) by (**a**) plasma synthesis, (**b**) Pt loading on BZ and TOAS.

A schematic of the experimental setup is illustrated in Fig. 1. A total volume of 200 ml of benzene (>99.5%, Kanto chemical) and thioanisole (>99%, Kanto chemical) was utilized, respectively, as the precursors of pristine carbon and S-doped carbon supports. The electric discharge was generated in each solution between high purity tungsten electrodes (99.999%, Nilaco Co. Ltd, a diameter of 1 mm) by using a bipolar pulse power supply (MPP-HV02, KURITA) operated at a voltage of ~2 kV, a frequency of 100 kHz, and a pulse width of 0.5 μs. Discharge in each solution was done for 20 min at a stable plasma state. The obtained carbon powder was separated from the solution by filtering through the polytetrafluoroethylene (PTFE; JVWP04700, Merck Millipore) membrane filter with 100 nm diameter. After that, it was washed and cleaned with ethanol. The washed carbon samples were dried in an oven at 100 °C for 24 h. The carbon supports synthesized from benzene and thioanisole are hereafter denoted as BZ and TOAS, respectively.

### Platinum loading on a carbon support

In a typical preparation, a glycol solution containing NaOH (50 mL, 0.5 M) was added into a glycol solution of H_2_PtCl_6_·6H_2_O (1.0 g, 1.93 mM in 50 mL), with stirring to obtain a transparent yellow platinum hydroxide solution, which was then heated at 160 °C for 3 h under an N_2_ atmosphere. A dark-brown opaque colloidal solution of Pt metal nanoclusters was obtained via heating. The Pt precursor solution (2 mL) was introduced into BZ and TOAS with dispersed ethanol solvent (30 mg in 50 mL) under pre-ultrasonication for 1 hour. Eventually, the powder was obtained via centrifugation and cleaning with ethanol (twice) before being dried in an oven at 70 °C for 24 h. To evaluate the effect of annealing on Pt nanoparticles, Pt/BZ and Pt/TOAS were annealed at 600 °C for 2 h under Ar flow. The Pt-loaded carbon supports are hereafter referred to as Pt/BZ and Pt/TOAS, and the samples annealed at 600 °C are named Pt/BZ_600 and Pt/TOAS_600.

### Structure analysis and chemical composition

Surface area and pore structures of carbon supports were analyzed using the nitrogen absorption-desorption method (BET Brunauer Emmett Teller; Shimadzu, TriStar-II3020). Morphology and chemical composition of the synthesized carbon materials were characterized using transmission electron microscopy (TEM; JEOL, TEM-2100/HR), X-ray diffraction (XRD; Rigaku, Ultima IV), scanning electron microscopy (SEM; JEOL, JSM-7100F), and X-ray photoelectron spectroscopy (XPS; JEOL, JPS-9010MC). Thermal stability was evaluated by thermal gravimetric analysis (TGA; Shimadzu, TA-60WS) from room temperature to 1300 °C at an elevation rate of 10 °C /min under an air atmosphere. Prior to the thermal stability test, Pt/BZ and Pt/TOAS were annealed at various temperatures (400, 500, and 600 °C) for 2 h under Ar flow.

### Electrochemical measurements

Electrochemical properties of the as-prepared Pt-loaded carbon materials (Pt/BZ, Pt/TOAS) were investigated using an electrochemical analyzer (704ES, BAS Inc.). For comparison purpose, a commercial 20 wt.% Pt/C (Vulcan XC-72) was applied as a benchmark ORR catalyst. The catalyst ink for the electrochemical measurements were prepared by dispersing 5 mg of finely ground catalyst in a mixture containing 480 μL of ethanol, 480 μL of distilled water and 40 μL of Nafion®117 solutions and ultrasonication for 1 hour. The catalyst loading was calculated to be 120 µg/cm^2^. Cyclic voltammetry (CV) was conducted using a three-electrode system. A total of 4 μL of well-dispersed catalyst ink was applied onto a freshly polished GC disk (diameter: 4.0 mm) surrounded by a Pt ring (inner/outer-ring diameter: 5.0/7.0 mm) electrode as a working electrode. A platinum coil and Ag/AgCl (saturated KCl) were used as the counter and reference electrodes, respectively. The CV measurements were performed in 0.1 M KOH saturated O_2_ at a scan rate of 50 mV s^−1^, and the potential ranges were measured from −1.0 V to 0.2 V.

## Results

### Proposed mechanisms of pristine and S-doped carbon (TOAS) generation via the plasma process

A schematic of the carbon formation mechanism via plasma synthesis is illustrated in Fig. 1a. Plasma chemistry during the carbon synthesis process has been discussed in previous studies. Li *et al*. described the mechanism of carbon synthesis via the plasma process as follows: the organic precursors were ionized and then reacted with each other as highly active radicals in the plasma and at the plasma/gas interface. When plasma discharges under benzene, the major radicals were found to be ·C_2_ followed by ·CH^36^. Hyun *et al*. also discussed the formation of heteroatom carbon. In their study, nitrogen-doped carbon was fabricated by applying various types of C–N precursor under a similar plasma process^37^. Since the dissociation energy (293 kJ/ mol) of the C–N bond is lower than that of the C–C bond (346 kJ/ mol), the authors suggested that the formation of carbon nanosheets or nanoparticles was due to the breakage of the C–N bond. During these steps, carbon residue formed through the cleavage of the C–N bond, resulting in long carbon chains. The chains then underwent changes to form a graphite-like structure by intermolecular crosslinking of adjacent chains through dehydrogenation reactions. Based on previous studies, we could consider the plasma reaction under thioanisole (C_7_H_8_S) as follows: the bond dissociation energy of each chemical bond in ascending order is C–S (272 kJ/mol) < C–C (346 kJ/mol) < C–H (411 kJ/mol) < C=C (602 kJ/mol). Thus, the formation of carbon nanoparticles would be due to the breakage of the C–S bond. In addition, ·C_2_ and ·CH were also generated and assisted the growth of long carbon chains, eventually forming the S-doped carbon nanoparticles. Thereafter, the Pt nanoparticles were synthesized via the solution method and loaded onto the pristine and S-doped carbon supports, as illustrated in Fig. 1b.

### Properties of pristine and S-doped carbon (TOAS) supports

**Figure 2:**
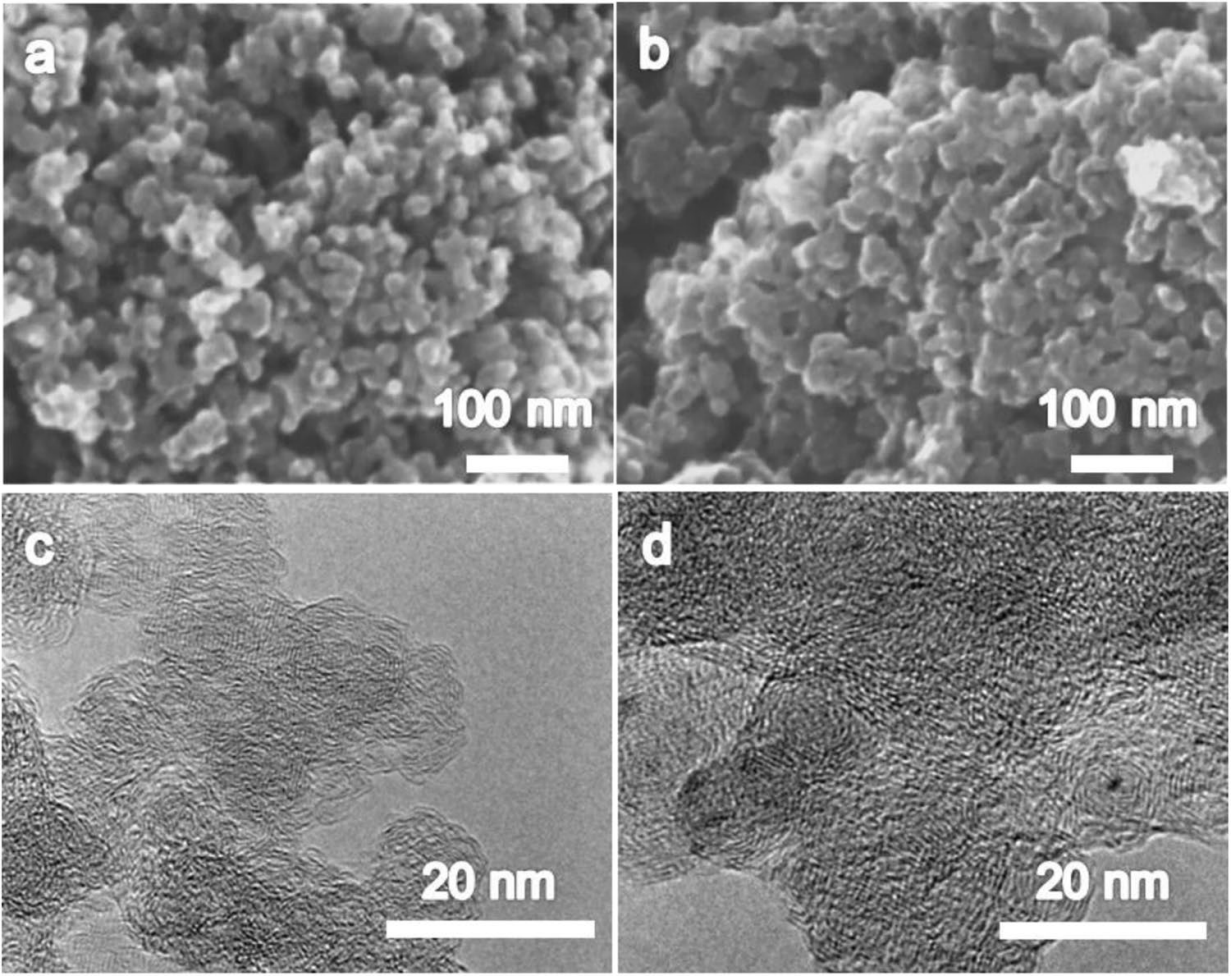
SEM images of (**a**) BZ, (**b**) TOAS, and TEM images of (**c**) BZ, and (**d**) TOAS.

The SEM and TEM morphologies of BZ and TOAS are shown in Fig. 2(a–d). The carbon support is formed of interconnected nanocarbon spheres with an average size of 10–20 nm. The carbon nanoparticles show disordered, short, and highly-bent graphitic layers, which can be categorized as turbostratic structures. These tiny, short graphitic layers were aggregated during the plasma process and formed as imperfect spheres, like carbon black. No significant differences in morphology were observed between BZ and TOAS. Contrarily, sulfur atoms in TOAS are evenly distributed within the carbon supports, based on the bright-field STEM image of TOAS (Fig. S1(a–d)). The sulfur in TOAS was approximately 4 wt.%, which was identified when thioanisole was applied as a precursor, and the sulfur group could then be retained within the carbon matrix and successfully form S-doped carbon supports. Table 1 summarizes the surface area, pore size and pore volume of BZ and TOAS, estimated by BET (Brunauer Emmett Teller) absolution-desorption measurements. Textual properties such as the size distribution of micropores (<10 nm), mesopores (10 nm~50 nm) and macropores (>50 nm) are also evaluated. The total surface area (SBET) and micro surface area (SMicro) and meso/macro surface area (Smeso/Macro) of BZ and TOAS are correspondingly 239 and 213 m^2^/g, 65 and 57 m^2^/g, and 174 and 156 m^2^/g. In terms of pore distribution, the percentage of mesopores and macropores in BZ and TOAS are, 72.8 and 73.2%, respectively. BZ exhibited a slightly higher surface area, but both carbon supports consisted of more micropores. The average pore size in BZ is 5.7 nm, which is smaller than that of TOAS (6.4 nm). However, the total pore volume of BZ and TOAS is similar, which is between 0.40–0.41 m^3^/g. From the above results, it can be concluded that the S-doping within the carbon matrix in TOAS has no significant effect on physical properties.

**Table 1:** Textural parameters of BZ and TOAS derived from N_2_ adsorption-desorption isotherms.

Sample	^*a*^ *S*_*BET*_ (m^2^ g^−1^)	^*b*^ *S*_*micro*_ (m^2^ g^−1^)	^*c*^ *S*_*meso/macro*_ (m^2^ g^−1^)	*S*_*meso/macro*_ */S*_*BET*_ (%)	^*d*^ *V*_*total*_ (m^3^ g^−1^)	^*e*^ *D* (nm)
BZ	239	65	174	72.8	0.41	5.7
TOAS	213	57	156	73.2	0.40	6.4

^a^Specific surface area determined by the multiple-point BET method using the adsorption branch of the isotherm in the *P/P*_*o*_ range of 0.05–0.30.

^b^Micro surface area determined by the *t*-plot method.

^c^External surface area (*S*_*meso/macro*_ = *S*_*BET*_ − *S*_*micro*_).

^d^Total pore volume determined by the BJH desorption branch of the isotherm between 1.7 nm and 300 nm.

^e^Average pore size determined by the BJH method using the desorption branch of the isotherm.

### Physical properties of Pt/BZ and Pt/TOAS before and after sintering

**Figure 3:**
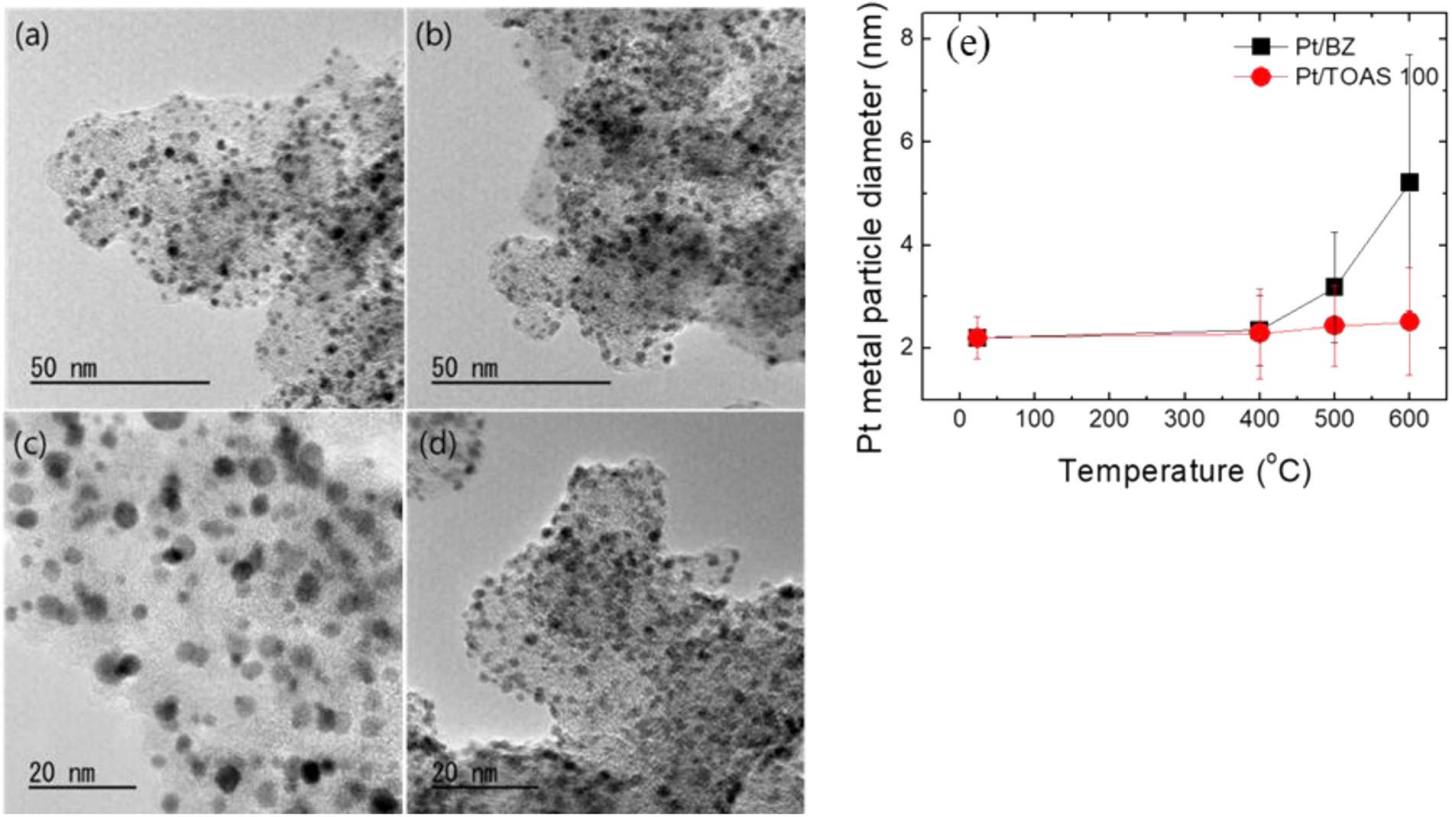
(**a**) TEM images of (**a**) Pt/BZ, (**b**) Pt/TOAS, (**c**) Pt/BZ_H600, (**d**) Pt/TOAS_H600 and (**e**) Size distribution of Pt nanoparticles (nm) in annealing temperature increased from 400 to 600 °C in Pt/BZ and Pt/TOAS.

High-resolution TEM (HRTEM) images of Pt/BZ, Pt/TOAS, Pt/BZ_600, and Pt/TOAS_600 are illustrated in Fig. 3. Figure 3(a) and (b) show that Pt NPs are highly dispersed on both carbon supports after Pt-loading procedures. The bright-field STEM image and energy dispersive X-ray spectrometer (EDS) spectrum of as-synthesized Pt/BZ and Pt/TOAS are shown in Fig. S2(a,b), respectively. The images show evenly distributed Pt nanoparticles and similar Pt loading on both carbon supports. The size range of as-prepared Pt NPs is 2.2 ± 0.40 nm in Pt/BZ and Pt/TOAS, which indicates that the carbon support has no effect on the size and distribution of Pt NPs. After annealing at 600 °C, the nanoparticles of Pt/BZ_600 in Fig. 3(c) appeared to exhibit a relatively larger size compared to that of Pt/TOAS_600 in Fig. 3(d). The size distributions of Pt NPs of as-prepared samples, and annealed samples at 400, 500 and 600 °C are averaged from TEM images and are plotted in Fig. 3(e). The Pt nanoparticles supported on Pt/BZ increased in sizes of 2.4, 3.2, and 5.2 nm after heat treatments at 400, 500, and 600 °C, respectively. Meanwhile, the average size of Pt NPs in Pt/TOAS increased only from 2.2 to 2.51 nm after annealing at 600 °C. The calculated increasing ratio of particle size is defined as follows:

$$[{D}_{afterthermaltreatmentat{600}^{^\circ }C}-{D}_{as \mbox{-} prepared}]/{D}_{as \mbox{-} prepared}\times 100$$

where *D* is the Pt particle diameter.

The calculated increase ratios of Pt NPs in Pt/BZ and Pt/TOAS were 137 and 14%, respectively (Table 2). The agglomeration of Pt NPs in Pt/BZ_600 can be further confirmed by bright-field STEM in Fig. S3(a). On the contrary, Fig. S3(b) exhibited uniformly dispersed Pt NPs in Pt_TOAS_600.

**Table 2:** Changes in the sizes of Pt nanoparticles after thermal treatments at different temperatures.

Sample	As-prepared (nm)	400 °C (nm)	500 °C (nm)	600 °C (nm)	Increasing ratio (%)^a^
Pt/BZ	2.2 ± 0.40	2.4 ± 0.68	3.2 ± 1.07	5.2 ± 2.49	137
Pt/TOAS	2.2 ± 0.40	2.3 ± 0.88	2.4 ± 0.78	2.5 ± 1.04	14

^a^The increasing ratios of particle size after heat treatment at 600 °C, estimated using the following equation.

**Figure 4:**
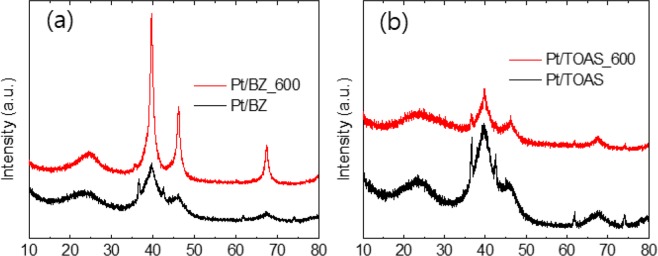
XRD analyses of (**a**) Pt/BZ and Pt/BZ_600, (**b**) Pt/TOAS and Pt/ TOAS_600.

The X-ray diffraction patterns of Pt/BZ and Pt/TOAS before and after annealing are compared in Fig. 4(a,b). All samples demonstrate a broad peak at 23.4° which is attributed to 002 of graphite in the turbostratic phase. Another major peak, between 38–45°, is composed by a board peak from graphite (101) at 43.2° and a sharp peak from Pt (111) at 39.7°. Other sharp peaks, at 46.1° and 67.5°, correspond to Pt (200) and (220), respectively. Small, sharp peaks corresponding to tungsten carbide (WC_1−x_) are also detected at 2θ ar36.7, 42.4, 61.8, and 74.2° corresponding to 111, 200, 220 and 311 reflections, respectively. These tungsten carbides were generated by the erosion or sputtering of the tungsten electrode during the plasma synthesis of carbon nanoparticles^38^. Based on the results of SEM-EDX, the amounts of tungsten carbide within the catalysts were less than 0.1 at.%, which is negligible compared to that of Pt NPs (6.2–8.3%). The average particle size of the Pt NPs could be calculated based on the broad peak areas from the XRD pattern using Scherrer’s formula d = 0.9 λ/βcos θ, where 0.9 is the shape factor generally taken for a cubic system, λ is the X-ray source wavelength, which is typically 0.154 nm, β is the full width at half maximum intensity in radians, and θ is the Bragg angle^39^. The average crystallite sizes for the Pt NPs before and after annealing are summarized in Table 3. The average size of Pt NPs in as-prepared Pt/BZ and Pt/TOAS are between 2.2–2.9 nm. After annealing at 600 °C, the average size of Pt NPs is larger than 6 nm in Pt/BZ_600 but remains less than 3.5 nm in Pt/TOAS. This result agrees with the TEM observations and thus proves that the S-doped carbon could resist agglomeration of Pt NPs. This sintering-resistance of Pt metal particles on TOAS is related to the doped sulfur’s strong affinity against heavy metals^40–42^. This strong chemisorption acts as an anchor between heavy metals and sulfur (or sulfur species). Without these anchor-bindings, Pt nanoparticles would be diffuse and aggregate after heating, which causes a larger particle size.

**Table 3:** The particle size of Pt NPs in various carbon catalysts (based on calculation from XRD).

Sample	Crystallite size (nm)		
	Pt (111) (2θ at 39.7°)	Pt (200) (2θ at 46.1°)	Pt (220) (2θ at 67.5°)
Pt/BZ	2.1	2.5	2.9
Pt/BZ_600	6.5	6.8	7.3
Pt/TOAS	2.2	2.4	2.7
PT/TOAS_600	2.8	3.1	3.4

### Chemical bonding states of Pt/BZ and Pt/TOAS before and after sintering

**Figure 5:**
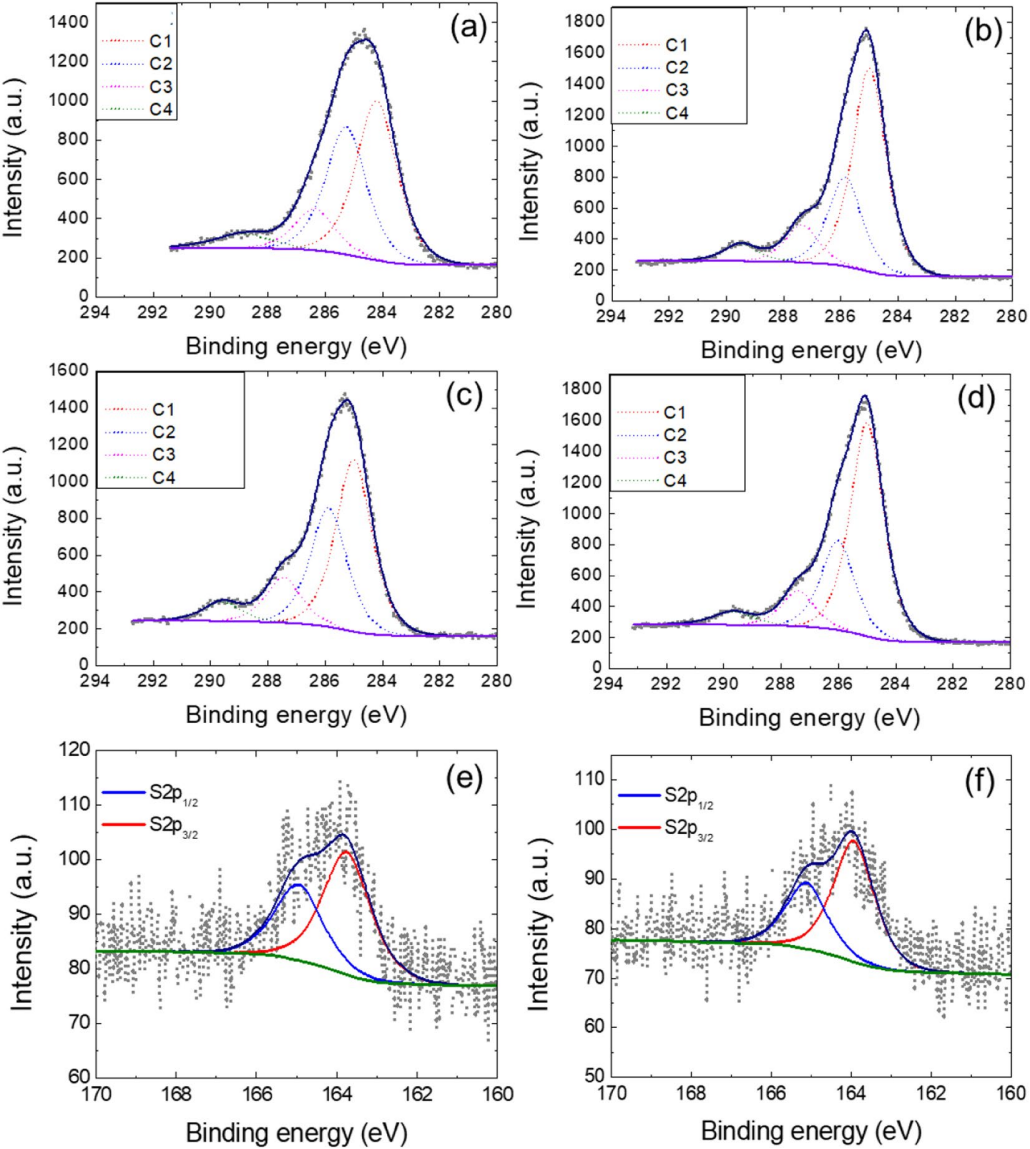
(**a**) High resolution C 1*s* XPS spectra with peak deconvolution of Pt/BZ, (**b**) Pt/BZ_600 and (**c**) Pt/TOAS, (**d**) Pt/ TOAS_ 600, and (**e**) S 2p XPS spectra with peak deconvolution of Pt/TOAS and (**f**) Pt/ TOAS_ 600.

X-ray photoelectron spectroscopy (XPS) was performed in order to understand the changes in chemical bonding states of Pt/BZ and Pt/TOAS before and after annealing. Detailed scans of C 1*s* and their deconvolutions of Pt/BZ, Pt/TOAS, Pt/BZ_600, and Pt/TOAS_600 are shown in Fig. 5(a–d). The C 1*s* reveals a dominant contribution of sp2 graphite-like carbon (C1) in an aromatic environment (285.00 eV), followed by C–O or C–S bonding (C2) at 286.06 eV. Also, minor contributions to the XPS signal at a binding energy of around 287.16 eV and 289.66 eV, respectively, correspond to the C toO (C3) or O  (C–O (C4) bonds. Carboxylic groups are formed during the oxidation of the carbon surface, which is common for a carbon catalyst^43^. Overall, no significant difference was observed between Pt/BZ and Pt/TOAS, although a slightly higher ratio of C2 could be observed in Pt/TOAS due to the presence of C–S bonding. After the annealing process at 600 °C, both carbon samples exhibited a higher ratio of C1, shown in Fig. 5(c,d). This could be explained on the basis of the relationship between the annealing temperature and the development of a crystalline alignment in sp2 graphite-like carbon during graphitization^44^. Figure 5(e,f) show detailed studies on sulfur bonding in Pt/TOAS and PT/TOAS_600. Carbon-sulfur (C–S–C) bonding can be divided into two relevant peaks for S 2*p*_1/2_ and S 2*p*_3/2_ at a binding energy of around 164.9 eV and 163.7 eV, respectively. On the contrary, oxidized sulfur species, such as sulfonate and sulfate groups at binding energies between 165.6 eV and 168. 8 eV, is hardly detected in the S 2*p* spectrum^45^. The results indicate that the sulfur atoms are doped within the carbon matrix. Also, there are no significant changes in ratio or atomic percentage in sulfur after the annealing process, which indicates that the S-doped carbon is thermally stable and that sulfur atoms are not likely to leave the carbon matrix at 600 °C. The peak assignments and the fraction of various bonds are summarized in Table 4.

**Table 4:** Peak assignments (at.%) for the C 1*s* and S 2*p* photoelectron envelopes for Pt/BZ, Pt/BZ_600, Pt/TOAS and Pt/TOAS_600.

Peak	Pt/BZ	Pt/BZ_600	Pt/TOAS	Pt/TOAS_600
C1 (sp^2^ C–C or C–H)	45.7	58.2	47.9	60.0
C2 (C–O/C–S)	36.7	25.8	33.5	25.3
C3 (C=O)	11.9	10.8	12.7	10.1
C4 (O=C–O)	5.7	5.2	5.9	4.6
**S1** (**Thiophenic C-S-C**)				
S 2*p*_1/2_	—	—	36.3	43.1
S 2*p*_3/2_	—	—	63.7	56.9

### Electrochemical characterization of Pt/BZ and Pt/TOAS

**Figure 6:**
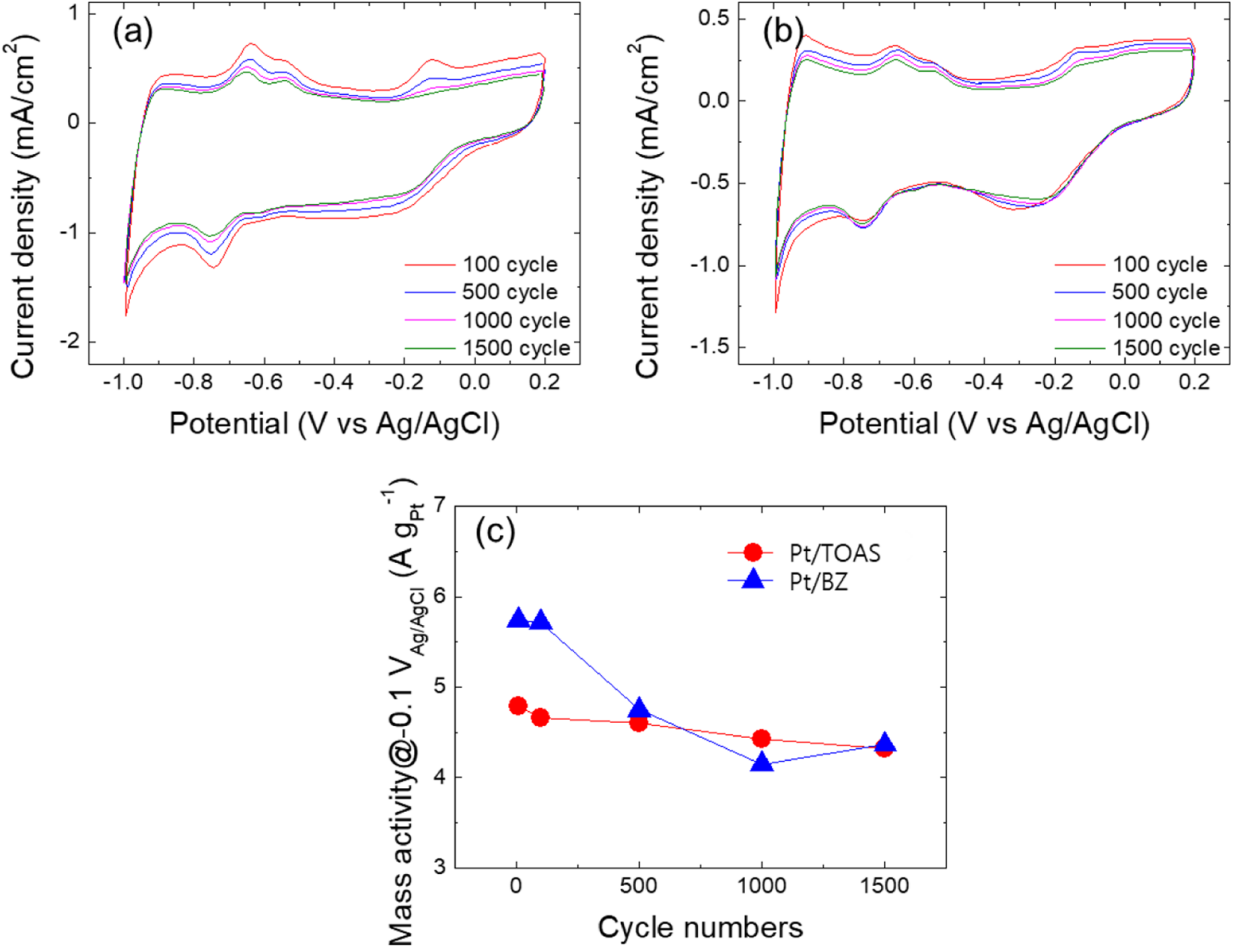
(**a**) Cyclic voltammograms (**a**) Pt/BZ, (**b**) Pt/ TOAS in 0.1 M KOH from 100 to 1,500 cycles, and (**c**) Calculated mass activity of Pt in Pt/BZ and Pt/TOAS at −0.1 V_Ag/AgCl_ (A g_Pt_^−1^).

The cyclic voltammograms (CVs) polarization plots, from 100 to 1500 cycles of accelerated stress test (AST) in O_2_-saturated 0.1 M KOH solutions at a scan rate of 50 mV s^−1^, are demonstrated for Pt/BZ and Pt/TOAS in Fig. 6(a,b), respectively. Both catalysts have an ORR onset potential at −0.017 (V vs Ag/AgCl) which is similar to that of fresh 20 wt. % Pt/C catalyst (Fig. S4(a)). However, the current density of Pt/BZ and 20 wt. % Pt/C decrease gradually with higher cycles and the current density of Pt/TOAS remains relatively stable.

In order to further estimate the stability of Pt nanoparticles, the mass activity of Pt/BZ and Pt/TOAS was evaluated at 0, 50, 500, 1000 and 15,000 cycles. The Pt loading of 20 wt.% Pt/C, Pt/BZ and Pt/TOAS was calculated as 72 μg_pt_ /cm^2^. The calculated kinetic current density, *i*, was normalized by Pt loading amounts on the glassy carbon to obtain the mass activity of Pt:

$$1/i=1/{i}_{k}=1/{i}_{l}$$

where *i*, *i*_*k*_, and *i*_*l*_ are the current density, kinetic current density, and limit current density, respectively.

The mass activity of Pt/BZ and Pt/TOAS with respect to cycle numbers is plotted in Fig. 6(c). The initial mass activity of the Pt/BZ catalyst at 0.1 V_Ag/AgCl_ was 5.8 A g_pt_^−1^, which is higher than that of Pt/TOAS (4.9 A g_pt_^−1^). The difference in the original mass activity between Pt/BZ and Pt/TOAS can be explained by differences in the resistivity of the carbon support. In general, the electrical properties of carbon are highly related to the structure and bonding of its matrices. Li *et al*. suggested that the resistivity of as-prepared carbon generated *via* the plasma process was highly related to the amount of sp^3^-hybridized hydrogen atoms that remained in the aromatic structure. The resistivity of the as-prepared carbon reduced by almost a hundred when the percentage of hydrogen in the carbon matrices decreased from 20.7 to 11.5 mol.%^46^. Hyun *et al*. also reported that the resistivity of N-doped carbon synthesized with N-methyl-2-pyrrolidone and (C_5_H_9_NO) and of 2-pyrrolidone (C_4_H_7_NO) was, respectively, 0.065 and 0.053 Ω∙cm^37^. If we consider the structure of benzene (C_6_H_6_) and thioanisole (C_6_H_5_SCH_3_), benzene only consisted of sp^2^-hybridized hydrogen, where thioanisole is composed of both sp^2^ and sp^3^-hybridized hydrogen. Since the presence of sp^3^-hybridized hydrogen increases resistivity, we can easily propose that the BZ has less resistivity compared to that of TOAS. The CV polarization plot also agrees with the above discussion, as the current density of Pt/BZ in Fig. 6(a) is higher compared to that of Pt/TOAS in Fig. 6(b). From Fig. S4(a,b), the commercial 20 wt% Pt/C has much higher conductivity and thus the mass activity is also higher by 1 magnitude.

According to Fig. 6(c), the mass activity of Pt/BZ decreased by 15% (~4.9 A g_pt_^−1^) where Pt/TOAS remained similar to its original mass activity (~4.8 A g_pt_^−1^) after 500 cycles. The mass activity of Pt/BZ gradually reduced further to 4.3 A g_pt_^−1^ after 1500 cycles, resulting in a total decrease to approximately 25% of its original mass activity. In Fig. S3(b), the mass activity of 20 wt.% Pt/C sharply reduces ~25% after 500 cycles and becomes relatively stable between 500–1500 cycles. This phenomenon is similar to that of Pt/BZ, as the unstable and small Pt NPs tends to migrate and agglomerate at the beginning of the test. After small Pt NPs agglomerate and grow larger, the bigger Pt NPs is relatively stable and the mass activity does not further reduce. On the other hand, the mass activity of Pt/TOAS reduces by less than 10% after 1500 cycles. The results illustrate clearly that sulfur atoms (thiol-) in TOAS acted as the capping agents for Pt nanoparticles. In the platinum-carbon supports, strong interactions between the sulfur atoms and the surface atoms of the loaded Pt nanoparticles play an important role in stabilizing the Pt particles against Ostwald ripening, and thus improved the stability of loaded Pt in longer cycles^18^.

## Conclusions

In this study, we firstly synthesized pristine carbon and sulfur-doped carbon nanoparticles as support materials via an SP process, and then loaded platinum (Pt) nanoparticles onto the pristine (Pt/BZ) and S-doped carbon matrix (Pt/TOAS). The sulfur content in TOAS was approximately 4 wt.% and was evenly distributed within the S-doped carbon support. From a sintering test, the Pt nanoparticles supported on pristine carbon (Pt/BZ) increased in sizes of 2.4, 3.2, and 5.2 nm after heat treatments at 400, 500, and 600 °C, respectively. Meanwhile, the average size of Pt NPs supported on S-doped carbon (Pt/TOAS) increased from only 2.2 to 2.51 nm after annealing at 600 °C. The chemical stabilization of Pt/TOAS under alkaline conditions was also investigated using cyclic voltammetry (CV) tests from 0 to 1500 cycles. The initial mass activity of the Pt/BZ catalyst at 0.1 V_Ag/AgCl_ was 5.8 A g_pt_^−1^, which was higher than that of Pt/TOAS (4.9 A g_pt_^−1^). After 500 cycles, the mass activity of Pt/BZ decreased by 15% (~4.9 A g_pt_^−1^) and then gradually reduced to 25% (~4.3 A g_pt_^−1^) after 1500 cycles. A similar trend was observed in commercial 20 wt.% Pt/C. On the contrary, the mass activity of Pt in Pt/TOAS reduced by less than 10% after 1500 cycles. The results illustrate clearly that sulfur atoms (thiol-) in TOAS stabilized Pt nanoparticles by a strong interaction between the sulfur atoms and the surface atoms of the loaded Pt nanoparticles, and thus improved the stability of loaded Pt in longer cycles. The enhanced stability of Pt nanoparticles by S-doped carbon supports is essential for the development of high-performance catalysts for metal-air batteries.

## Supplementary information

supplmentary file
