# Supplementary material for: Enhanced Electrocatalytic Stability of Platinum Nanoparticles Supported on Sulfur-Doped Carbon using in-situ Solution Plasma
Source: Sci Rep. 2019 Sep 3;9:12704. doi: 10.1038/s41598-019-49194-x (PMC6722099; doi:10.1038/s41598-019-49194-x)
Supplement: Supplementary file 1 — supplmentary file [file 41598_2019_49194_MOESM1_ESM.docx]

Enhanced Electrocatalytic Stability of Platinum Nanoparticles Supported on Sulfur-Doped Carbon using *in-situ* Solution Plasma

Oi Lun Li^a^, Zhicong Shi^b^ , Hoonseung Lee^c*^ and Takahiro Ishizaki^d^*

^a^ School of Materials Science and Engineering, Pusan National University, Busan, 46241, Korea

^b^ School of Materials and Energy, Guangdong University of Technology, Guangzhou, 510006, China

^c^ Energy Environment Center Fusion Energy Team, Korea Marine Equipment Research Institute, Busan, 49111, Korea.

^d^ Department of Materials Science and Engineering, College of Engineering, Shibaura Institute of Technology, Tokyo, 135-8548, Japan

***** Corresponding author: +81-3-5859-8115, [ishizaki@shibaura-it.ac.jp](mailto:ishizaki@shibaura-it.ac.jp)

**Supplementary data**


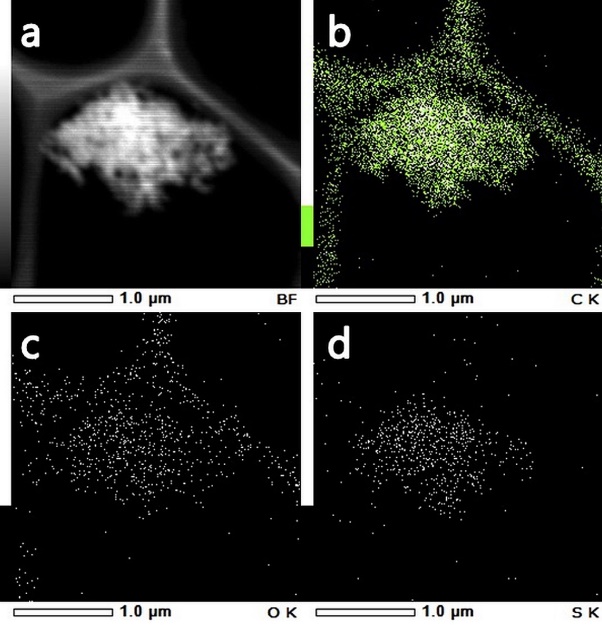


**Figure S1**. (a) Bright-field STEM image of TOAS, (b) carbon, (c) oxygen and (d) sulfur corresponding to EDS elemental mapping of (a)


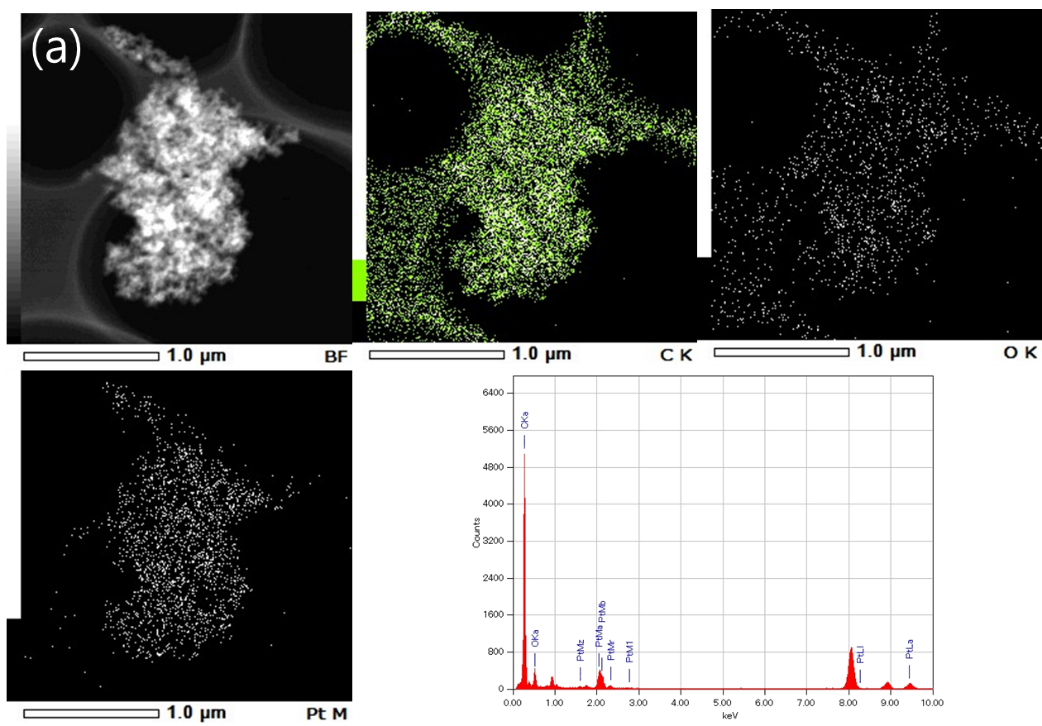


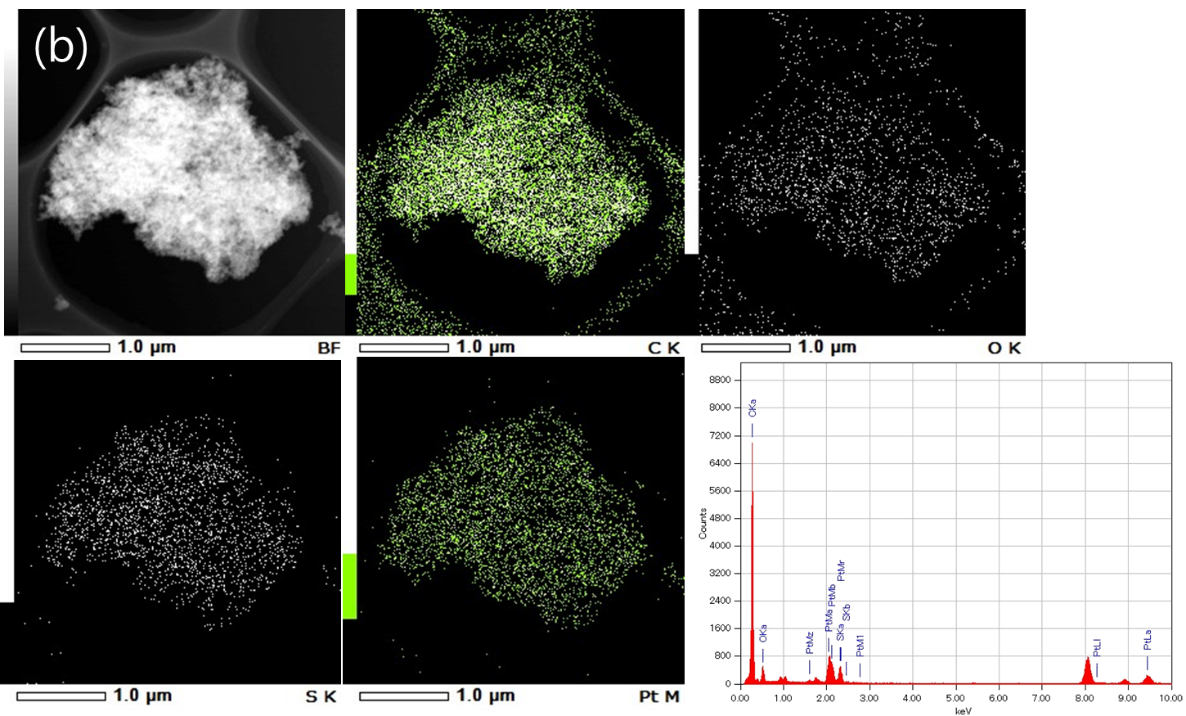


**Figure S2**. Bright-field STEM image and energy dispersive X-ray spectrometer (EDS) spectrum of as-synthesized (a) Pt/BZ and, (b) Pt/TOAS


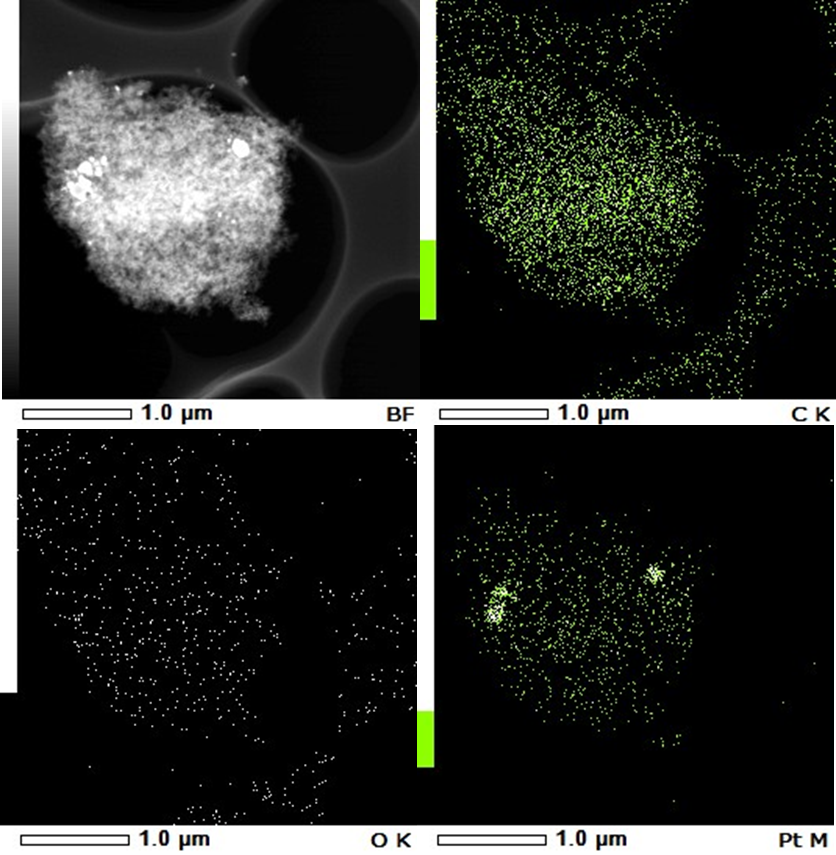

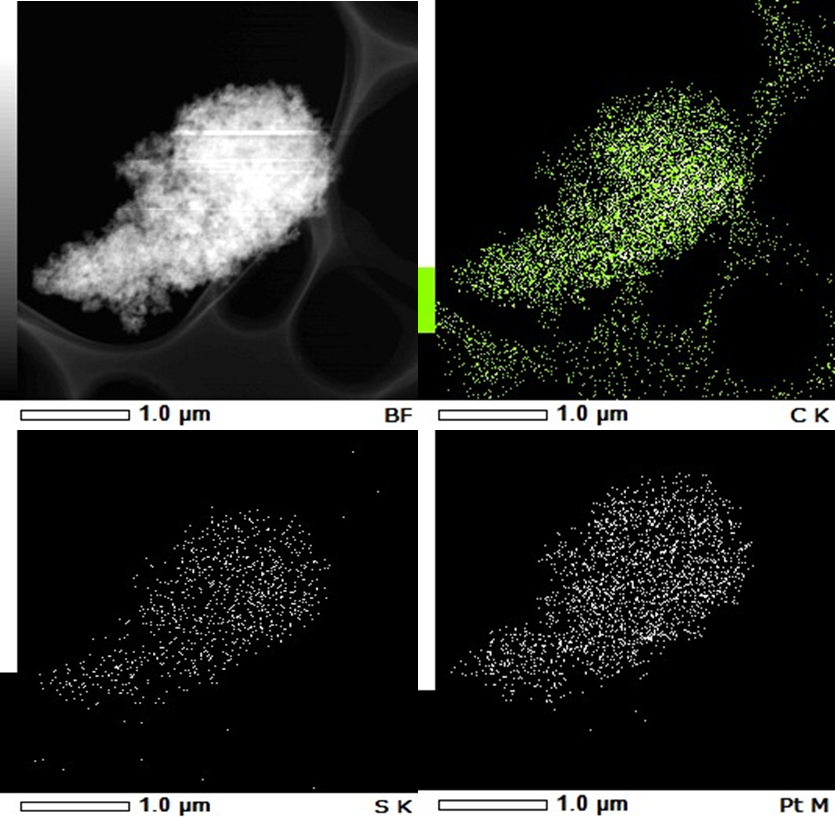


1. (b**)**

**Figure S3**. Bright-field STEM image corresponding to EDS elemental mapping of (a) Pt/BZ_600 and, Pt/TOAS_600


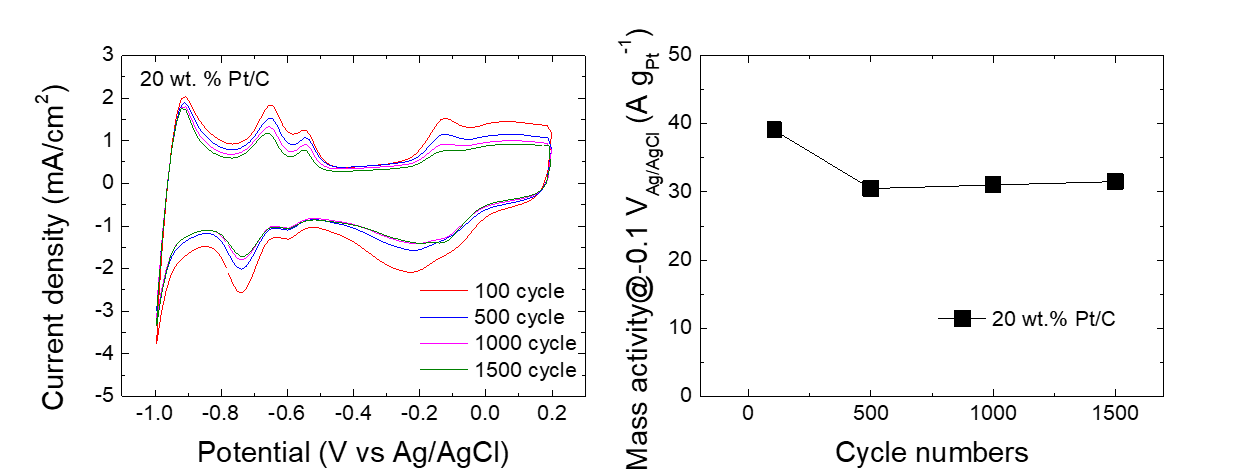


**Figure S4**. Electrochemical anlayses for commercial 20 wt.% Pt/C: (a) Cyclic voltammograms in 0.1 M KOH from 100 to 1,500 cycles, and (b) Calculated mass activity of Pt at -0.1 VAg/AgCl ( A gPt^-1^)
